# Irisin deletion induces a decrease in growth and fertility in mice

**DOI:** 10.1186/s12958-021-00702-7

**Published:** 2021-02-13

**Authors:** Yunyao Luo, Xiaoyong Qiao, Yaxian Ma, Hongxia Deng, Charles C. Xu, Liangzhi Xu

**Affiliations:** 1 Reproductive Endocrinology and Regulation Laboratory West China Second University Hospital, Sichuan University, #20 Section 3, Ren Min Nan Road, Chengdu, 610041 Sichuan China; 2 The Joint Laboratory for Reproductive Medicine of Sichuan University–The Chinese University of Hong Kong, Chengdu, People’s Republic of China; 3 Key Laboratory of Birth Defects and Related Diseases of Women and Children (Sichuan University) Ministry of Education, Chengdu, People’s Republic of China; 4 Department of Obstetrics and Gynecology, West China Second University Hospital, Sichuan University, Chengdu, People’s Republic of China; 5 College of Engineering, The Ohio State University, Columbus, OH USA

**Keywords:** Irisin, Growth and development, Hormone metabolism, Reproduction

## Abstract

**Background:**

Irisin, which is cleaved from fibronectin type III domain-containing protein 5 (*Fndc5*), plays an important role in energy homeostasis. The link between energy metabolism and reproduction is well known. However, the biological actions of irisin in reproduction remain largely unexplored.

**Methods:**

In this study, we generated *Fndc5* gene mutation to create irisin deficient mice. Female wild-type (WT) and *Fndc5* mutant mice were fed with standard chow for 48 weeks. Firstly, the survival rate, body weight and fertility were described in mice. Secondly, the levels of steroid hormones in serum were measured by ELISA, and the estrus cycle and the appearance of follicles were determined by vaginal smears and ovarian continuous sections. Thirdly, mRNA-sequencing analysis was used to compare gene expression between the ovaries of *Fndc5* mutant mice and those of WT mice. Finally, the effects of exogenous irisin on steroid hormone production was investigated in KGN cells.

**Results:**

The mice lacking irisin presented increased mortality, reduced body weight and poor fertility. Analysis of sex hormones showed decreased levels of estradiol, follicle-stimulating hormone and luteinizing hormone, and elevated progesterone levels in *Fndc5* mutant mice. Irisin deficiency in mice was associated with irregular estrus, reduced ratio of antral follicles. The expressions of *Akr1c18*, *Mamld1*, and *Cyp19a1*, which are involved in the synthesis of steroid hormones, were reduced in the ovaries of mutant mice. Exogenous irisin could promote the expression of *Akr1c18*, *Mamld1*, and *Cyp19a1* in KGN cells, stimulating estradiol production and inhibiting progesterone secretion.

**Conclusions:**

Irisin deficiency was related to disordered endocrinology metabolism in mice. The irisin deficient mice showed poor growth and development, and decreased fertility. Irisin likely have effects on the expressions of *Akr1c18*, *Mamld1* and *Cyp19a1* in ovary, regulating the steroid hormone production. This study provides novel insights into the potential role of irisin in mammalian growth and reproduction.

**Supplementary Information:**

The online version contains supplementary material available at 10.1186/s12958-021-00702-7.

## Introduction

Reproduction and fertility are closely linked to energy metabolism and the endocrine function of the adipose tissue, as both obesity and insufficient weight are associated with infertility in both males and females [1]. Most feeding-related peptides in mammals and teleost’s have been implicated in the regulation of energy balance and reproduction [2]. Irisin was first identified as a myokine by Bostrom et al. in 2012. They demonstrated that irisin is produced by proteolytic processing of a transmembrane receptor. Fibronectin domain-containing protein 5 (*Fndc5*) is a 209-residue protein with an N-terminal 29-residue signal sequence followed by the irisin or putative fibronectin III domain, a linking peptide, a transmembrane domain, and a 39-residue cytoplasmic segment. Cleavage in the linking peptide region releases soluble irisin into the extracellular milieu [3]. Irisin is 100% identical between mice and humans and is capable of stimulating adipocyte browning and thermogenesis, which suggests that there is a strong relationship between energy expenditure and irisin [3]. Irisin is secreted from the skeletal muscle, subcutaneous tissue, visceral adipose tissue, liver, brain, testis and so on [4–6]. However, recent studies have shown that the ovaries and endometrium are also able to produce irisin [7].

More recent data suggest a relationship between irisin and sex hormone metabolism. In primary cultures of tilapia pituitary cells, irisin is effective in stimulating both LHβ and FSHβ mRNA expression in vivo and in vitro [8]. Exogenous administration of irisin was significantly stimulating LH levels in obese female mice [9]. Irisin can also stimulate granulosa cells to produce estradiol [10]. However, the possible effects of irisin on sex hormone metabolism are controversial. Some studies have demonstrated negative effects of irisin on the hypothalamus-pituitary-gonadal (HPG) axis. In male rats, the administration of irisin reduces testosterone levels by suppressing LH and FSH secretion [11, 12]. Some of the effects on sex hormones are known, but the reproductive effects of irisin remain largely unexplored. In humans, the systemic irisin level increases around the time of puberty onset [13] and increases significantly throughout pregnancy [14]. These results strengthen the hypothesis that irisin affects the HPG axis and the regulation of reproductive functions. To date, the reproductive function of irisin in females has not been investigated. In this study, we analyzed the phenotypic and molecular characteristics of irisin deficiency in female mice.

## Materials and methods

This study was approved by the Ethics Committee of West China Second University Hospital, Sichuan University, China.

### Reagents

Antibody against *Fndc5* and *Cyp19a1* were obtained from Abcam (Cambridge, MA, USA). Antibody against *Akr1c18* was purchased from the brand R&D Systems Bio-Techne (Minneapolis, MN, USA). Antibody against *Mamld1* was purchased from Invitrogen (Carlsbad, CA, USA). Antibodies against ERK, p-ERK, P38, p-P38 were purchased from Cell Signaling Technology (Waltham, MA, USA). Antibodies against *GAPDH*, *β-Actin* and HRP-conjugated secondary antibodies were purchased from Zen-Bioscience Company (Sichuan, China). The enzyme-linked immunosorbent assay (ELISA kit) for estradiol (E2), progesterone (P), testosterone (T), follicle stimulating hormone (FSH), luteinizing hormone (LH) were purchased from Elab-science Company (Wuhan, China). The ELISA kit for growth hormone (GH) and insulin-like growth factor I (IGF-1) were purchased from Cusabio (Wuhan, China). The Wright stain was purchased from Solarbio Company (Beijing, China). Beyond that, DMEM/F12 medium, fetal bovine serum (FBS), penicillin and streptomycin were purchased from Gibco (Grand Island, NY, USA).

### Cell culture

Frozen stocks of human ovarian granulosa (KGN) cell line were thawed and cells were plated in 10-cm dishes (1 × 10^6 cells/plate). Then the cells were cultured in DMEM/F12 medium (Gibco, Grand Island, NY, USA) supplemented with 10% FBS (Gibco), 100 units/ml penicillin and 100 μg/ml streptomycin at 37 °C in an atmosphere of 5% CO_2_. Four groups were divided: medium alone (control) and medium with 10 nM, 20 nM and 30 nM irisin. After 48 h cultured, cells were harvested for qRT-PCR and Western blotting.

### Irisin deficient mice

**Fig. 1:**
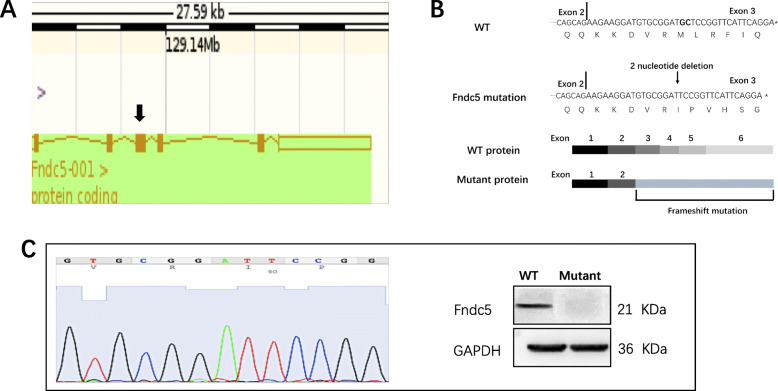
Generation of *Fndc5* mutation mice. a: Schematic representation of the gene targeting procedure. The black arrowhead indicates the target exon. b: Sequences of the *Fndc5* mRNA transcripts in mice and the proteins predicted to be encoded by the respective mRNAs. c: Confirmation of *Fndc5* mutation. Genotyping (left panel) and Western blot (right panel) analyses were consistent with successful *Fndc5* mutation

Irisin is cleaved from fibronectin type III domain-containing protein 5 (*Fndc5*). In this study, *Fndc5* mutation was performed to generate irisin deficient mice. C57BL/6 *Fndc5*-heterozygous (+/−) female mice were generated by View solid-biotech, Inc. (Beijing, China). Transcription activator-like effector nuclease (TALEN) technology was used to shear the DNA encoding the exon of the target gene. *Fndc5* has 6 exons, and the coding gene of irisin is located in exon 3. Clipping the 18th and 19th nucleotides in exon 3 led to a frameshift mutation, which induced irisin deficiency in mice (Fig. 1a, b). *Fndc5*-heterozygous (+/−) female mice were bred with C57BL/6 *Fndc5*-wildtype (+/+) (WT) male mice (Dossy, Chengdu, China) to produce heterozygous (+/−) male mice. Subsequently, female *Fndc5*-deficient(−/−)mice (Fig. 1c) were produced by mating heterozygous (+/−) female mice with heterozygous (+/−) male mice. PCR-based genotyping analysis with tail genomic DNA was performed for *Fndc5* using the following primers: 5′-CATGTTTCCTTAGCTCTACTGTG-3′ (forward) and 5′-GGAGAAAGCATGCATGGCAGTCT-3′ (reverse). There were 51 mutant mice in the experimental group, and 44 WT mice in the control group. Mice were housed with 4 to 5 animals per cage in a temperature-controlled environment on a 12-h light/dark cycle. All animals were given access to food (Dossy company, Chengdu, China) and water. To examine the effects of irisin in growth and reproduction, we compared the ovarian morphology, estrous cycle and circular hormone levels between two groups, at the age of 20 weeks. Besides, we recorded mouse mortality, body weight, and farrowing rate to from birth to 48 weeks.

### Expression and characterization of purified recombinant irisin

In this study, recombinant irisin was produced by *E. coli.* The expression and purification of irisin were performed as previously reported, with slight modifications [15]. We used the pET28M vector instead of the pET28a^+^ vector. Irisin can stimulate the phosphorylation of the p38 and ERK proteins in the 3 T3-L1 cell line. 3 T3-L1 cells (Bioleaf, Shanghai, China) were cultured with complete DMEM (Gibco, Grand Island, NY, USA) supplemented with 10% fetal bovine serum (Thermo Fisher Scientific, Waltham, MA, USA), 100 U/ml penicillin, and 100 μg/ml streptomycin (Thermo Fisher Scientific) at 37.0 °C in a humidified atmosphere of 95% air and 5% CO2. Briefly, 3 T3-L1 cells were cultured at a density of 2 × 10^5^ cells per 6-cm dish and treated with recombinant r-irisin or PBS (control) for 5, 10, 20, or 30 min. Then, the cells were harvested for Western blotting. As shown in Fig. 6a, phosphorylated p38 (P-p38) and phosphorylated ERK (P-ERK) levels were significantly increased at as early as 5 min, peaked between 10 and 20 min, and had decreased at 30 min after irisin treatment in 3 T3-L1 cells, while the total p38, ERK and GAPDH protein levels did not differ significantly among different times. The results indicated that irisin could activate downstream signaling pathways to exert bioactivity.

### RNA sequencing assay

mRNA-seq experiments were performed by Novogene (Beijing, China). An mRNA-seq library was prepared for sequencing using standard Illumina protocols. Briefly, total RNA was isolated from WT or mutant mouse ovaries, muscle or hypothalamus using TRIzol reagent (Invitrogen) and treated with RNase-free DNase I (New England Biolabs, MA, USA) to remove any contaminating genomic DNA. mRNA was purified from total RNA using poly-T oligo-attached magnetic beads (Invitrogen Dynal). Double-stranded complementary DNAs were synthesized using Superscript II reverse transcriptase (Invitrogen) and random hexamer primers. The cDNAs were then fragmented by nebulization, and the standard Illumina protocol was followed thereafter to create the mRNA-seq library. For data analysis, basecalling was performed using CASAVA. Reads were aligned to a genome using the split read aligners TopHat and Bowtie2 with default parameters. HTSeq was used for estimating abundances.

### Quantitative real-time PCR (qRT-PCR)

Total RNA was isolated with the Cell Total RNA Isolation Kit (Fore Gene, Inc. Chengdu, China) and then used for cDNA synthesis with RT EasyTM II (Fore gene, Inc., Chengdu, China) according to the manufacturer’s instructions. qPCR was performed using a PCR Easy TM kit (Fore Gene, Inc., Chengdu, China) and the Applied Biosystems 7500 Real-Time PCR System under the following conditions: 95 °C for 3 min, followed by 40 cycles of 95 °C for 15 s and 60 °C for 30 s. In addition, the comparative 2^-ΔΔCT^ method was adopted to analyze gene expression. *GAPDH* was used as the endogenous reference gene. Primers were generated using Primer 3 and were listed in Table 1. Meanwhile, all qRT-PCR assay was performed in triplicate.

**Table 1:** The relevant parameters of qRT-PCR

Gene	Primers	Accession number	Tm(°C)	qPCR efficiency(%)
GAPDH	F:5′-GGAGCGAGATCCCTCCAAAAT-3′	Nm_002046.7	60	95.2
	R:5′- GGCTGTTGTCATACTTCTCATGG-3′			
Fndc5	F:5′ -TGGTAATCCCTGGACTGCAG-3′	NM_153756.3	60	92.7
	R:5′- GGGTACAAGGAGATGGAGGG-3′			
Akr1c18	F:5′- ACCAAATTGGCAATTGAAGCT-3′	NM_134066.3	60	89.6
	R:5′- TGGGATCACTTCCTCACCTG-3′			
Mamld1	F: 5′-TTCTGCAGCAGATGATGCAG-3’	NM_005491.5	60	90.3
	R:5′- GAGGATCTTGCCTGCTAGTG-3’			
Cyp19a1	F:5′- ATGTGGACGTGTTGACCCTTCT-3’	NM_000103.4	60	91.5
	R:5′- AGGAGAGCT TGCCATGCATCAA-3’			

### Western blot

Ovarian tissue and KGN cells were washed with PBS, harvested and lysed using RIPA Lysis Buffer (Beyotime Inst Biotech, Shanghai, China). According to the manufacturer’s instructions, a bicinchoninic acid (BCA) protein assay kit (Pierce, IL, USA) was used to determine the protein concentrations of the tissue and cell lysates. In total, 40 μg of lysate protein were resolved by 12.5% SDS-PAGE. After electrophoresis, the protein was transferred to 0.22-μM polyvinylidene fluoride (PVDF) membranes. The membranes were incubated with 5% nonfat milk for 1 h at room temperature to block nonspecific binding sites and then incubated overnight at 4 °C with primary antibodies. After a thorough wash with TBST and incubation with HRP-conjugated secondary antibodies, the membranes were washed again and then incubated for 2–5 min in enhanced chemiluminescence reagent (Millipore, Billerica, MA, USA). The signals were detected using Clarity Western ECL Substrate (Bio-Rad). The optical density of each target protein was corrected using that of GAPDH or β-Actin and analyzed with Quantity One (Bio-Rad).

### SiRNA transfection

When the KGN cell density reached 50%, the cells were transfected with validated siRNAs specific for *Fndc5* at a concentration of 100 nM with the riboFECTTM CP Transfection Kit (RIBOBIO, Guangzhou, China). The *Fndc5* gene-specific siRNAs were purchased from RIBOBIO (Guangzhou, China), and the targeting sequences were as follows: si-*Fndc5*#1, GATGGCCTCCAAGAACAAA; si-*Fndc5*#2, GGTGTCATTGCCCTCTTCT; and si-*Fndc5*#3, GGAGGATACGGAGTACATA. After the KGN cells were incubated with the siRNAs for 48 h or 72 h, total RNA and protein were harvested for qRT-PCR and Western blot analyses, respectively.

### Vaginal lavage and estrous cycle determination

Estrous cycles were monitored in female mice (*Fndc5* mutant and WT) beginning at 12 weeks old. Vaginal cytology was assessed by dipping a sterile swab in water and gently swabbing the outer half of the vaginal canal. The vaginal samples were transferred to a microscope slide, air dried, stained using a Wright Stain solution, dehydrated, and then cover slipped prior to visualization with a light microscope. The mouse estrus cycle was evaluated by observing the relative proportions of epithelial nucleated cells, squamous cells and leucocytes in vaginal smears.

### Ovaries collection and follicles counting

All ovarian samples were free of oviduct, adipose, and bursal tissue. The ovaries from one side were immediately stored at − 80 °C until RNA extraction and protein quantification. The ovaries from the other side were fixed in paraformaldehyde solutions for serial sectioning and H&E staining. The ovaries (one side per mouse) were subjected to routine paraffin embedding and serial sectioning (6-μM thickness) throughout the entire ovary. The sections were adhered to slides and stained with H&E using standard procedures. Follicle populations were counted in every third section of the entire ovary and were scored as primordial, primary, secondary, antral, or Graafian follicles based on their morphological appearance, as detailed in the literature [16].

### Measurement of hormone levels

The levels of estradiol (E2), progesterone (P), testosterone (T), follicle stimulating hormone (FSH), and luteinizing hormone (LH) in serum samples were measured using ELISA kits (Elabscience, Wuhan, China). GH and IGF-1 concentrations were determined by using ELISA kits (CUSABIO, Wuhan, China). The ELISAs were performed according to the manufacturer’s instructions. Moreover, after KGN cells were stimulated with various concentrations of irisin, the levels of E2 and P in the culture medium were determined by using a radioimmunoassay kit (Xin Fan Biotechnology, Shanghai, China).

### Statistical analysis

All data are presented as the mean ± SD. The χ2 test was used to investigate the farrowing rate. A one-way analysis of variance (ANOVA) followed by Tukey’s test was used to determine the significance between experimental data. The repeated measures ANOVA was used to analyze the body weight over time. A value of *P* < 0.05 was considered statistically significant.

## Results

### Irisin deletion causes a high mortality rate and low body weight

**Fig. 2:**
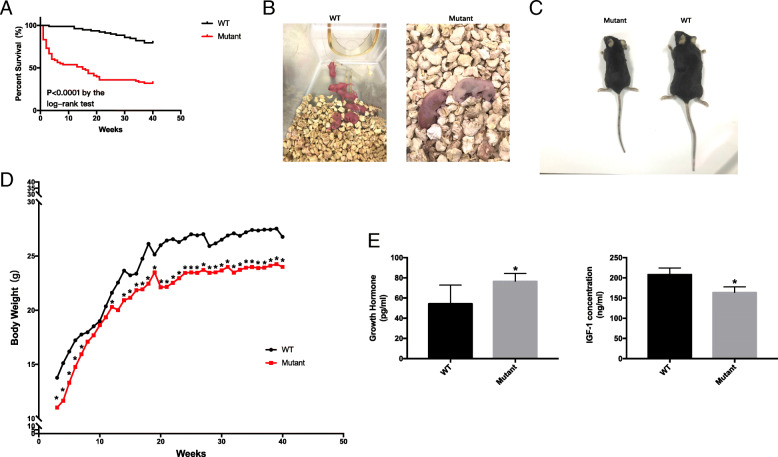
Irisin deletion influenced the survival and body weight of *Fndc5* mutant mice. a: The *Fndc5* mutant mice showed high mortality. Percentages of surviving WT (*n* = 44) and *Fndc5* mutant (*n* = 51) mice (*P* < 0.0001). b: Appearance of pups. c: Appearance of adult mice. d: Body weight (n_wt_ = 39_,_ n_mutant_ = 20). e, F: Growth hormone (GH) and insulin-like growth factor I (IGF-1) concentrations (*n* = 12 per group); *P < 0.05 compared to WT mice

Irisin deletion resulted in the death of 31 out of 51 female (~ 61% mortality) mutant mice (Fig. 2a, b), while the death rate was 18.5% (17/92) in heterozygous mice and only 11.4% (5/44) in WT mice (Table 2). Moreover, we weighed mice weekly from 3 weeks to 40 weeks of age and found that *Fndc5* mutant mice had significantly reduced body weights. At the age of 40 weeks, *Fndc5* mutant mice weighed 10.0% less than WT mice (24.11 ± 3.37 g vs 26.78 ± 4.31 g, respectively) (Fig. 2c, d). To identify the related causes of the elevated mortality rate and reduced body weight, we measured GH and IGF-1 levels in mice. We found that *Fndc5* mutant mice had a significantly higher GH level but a lower IGF-1 level than WT mice (76.55 ± 7.63 pg/ml vs 54.32 ± 18.44 pg/ml; 164.12 ± 5.10 ng/ml vs 208.01 ± 6.23 ng/ml, respectively; *P* < 0.05) (Fig. 2e).

**Table 2:** Survival and fertility of WT and Fndc5 mutant mice

Genotype	Total No.	Surviving No.	Pregnant No.	Farrowing rate (%)
WT	44	39	25	64.1
HT	92	75	44	58.7
FM	51	20	3	15.0^*^

WT: wildtype; HT: heterozygous;FM: Fndc5 mutant. Farrowing rate (%) = Pregnant number/Surviving number. *P < 0.05 compared to WT.

### Irisin deletion causes a low farrowing rate

By mating heterozygous parents for two years, we obtained twenty female *Fndc5* mutant mice and thirty-nine female WT mice that survived. We recorded mouse pregnancies and found that *Fndc5* mutant mice had a significantly lower farrowing rate (15.0%) than WT (64.1%) (*P* < 0.05) and HT mice (58.7%). There was no difference between the WT and HT groups (Table 1).

### Ovarian dysfunction in *Fndc5* mutant mice

#### Impaired estrous cycling following irisin deletion

**Fig. 3:**
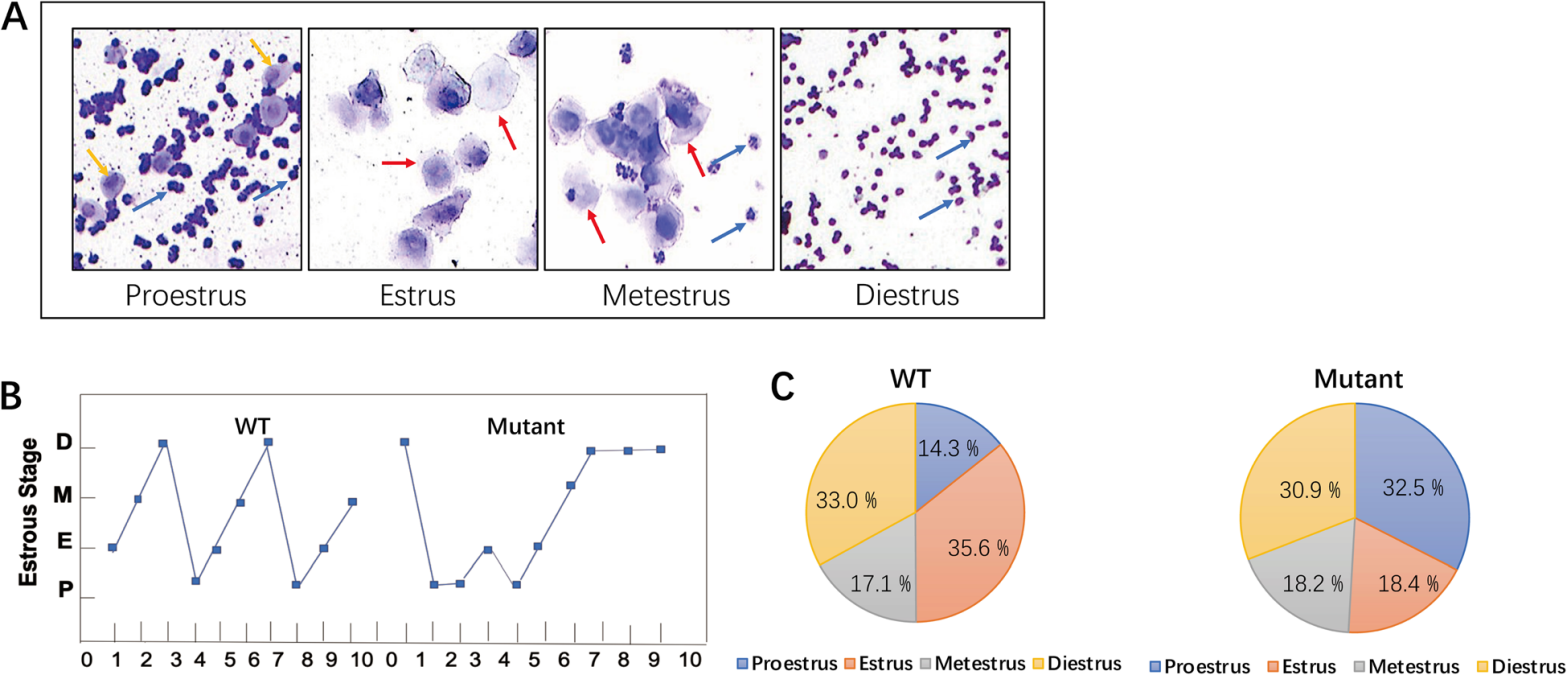
Irisin deletion affected the estrous cycle in *Fndc5* mutant mice. a: Example images of estrous cycle phases. Yellow, red, and blue arrowheads indicate epithelial nucleated cells, squamous cells, and leucocytes, respectively; 200x. b: Representative estrous cycle of the mice in each group (*n* = 10). c: The distribution of estrous cycle phases (n = 10). *P < 0.05 compared to WT mice

To determine whether irisin deficiency has an important impact on the onset of estrous cycling, we analyzed the vaginal cytology of mice. Representative images of vaginal cytology for each estrous cycle stage are shown in Fig. 3a, and the proportion of mice in each stage of the estrous cycle was also assessed. The results showed that *Fndc5* mutant mice displayed more irregular cycles than regular cycles, which were present in WT mice (Fig. 3b). The proestrus time of *Fndc5* mutant mice was prolonged compared to that of WT mice (32.5% vs 14.3%, *P* < 0.05); however, estrus was much shorter in mutant mice than in WT mice (18.4% vs 35.6%, P < 0.05) (Fig. 3c).

#### Appearance and histology of the ovaries

**Fig. 4:**
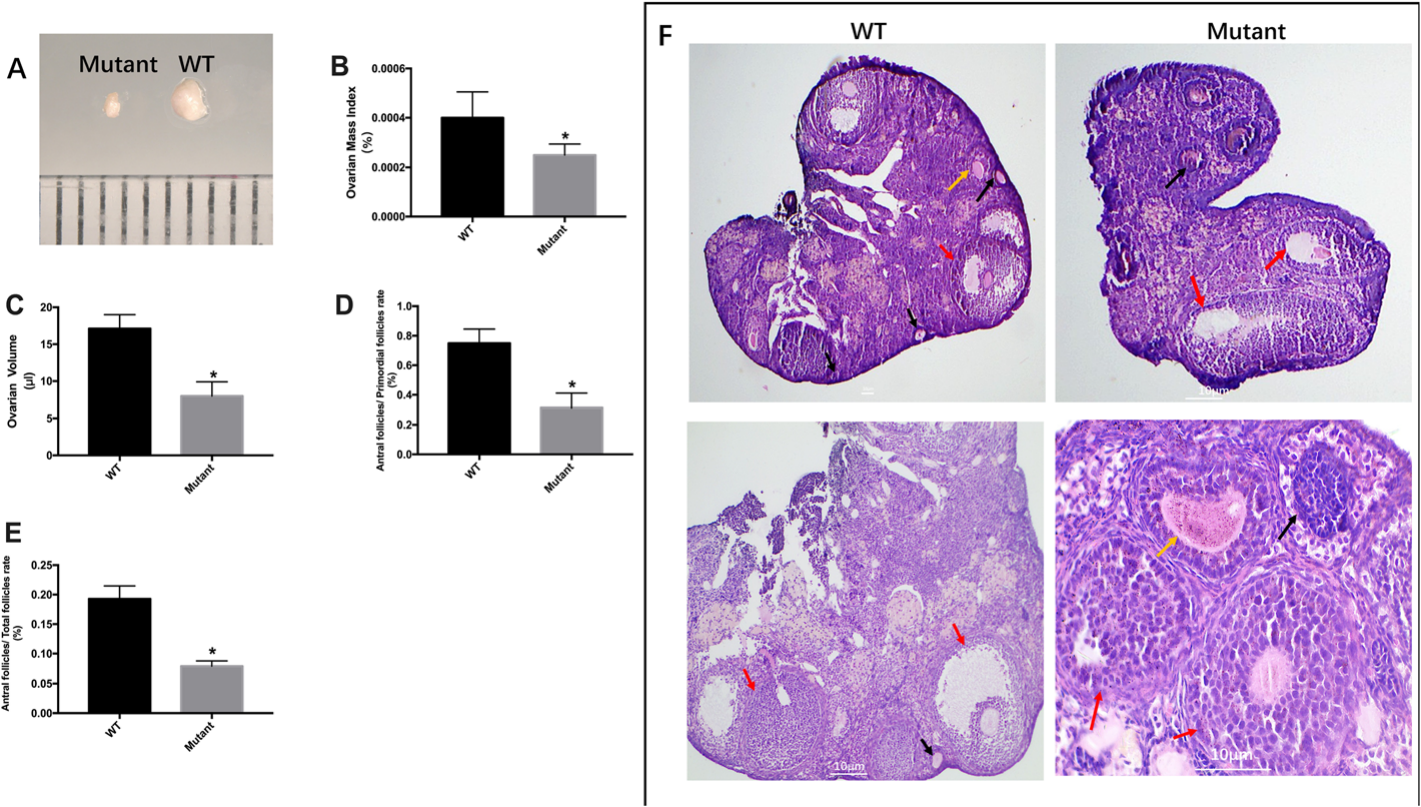
Ovary morphological analysis. a-c: Morphological findings for the ovaries. Ovarian mass index = ovary weight (g) / body weight (g); d: The primordial follicle to antral follicle ratio determined by in vivo ovarian analysis (n = 10). e: The antral follicle to total follicle ratio (n = 10). f: H&E staining of follicles in different stages in WT and *Fndc5* mutant mice. Black arrowheads, primordial follicle; yellow arrowheads, primary follicle; red arrowheads, antral follicle. *P < 0.05 compared to WT mice

Our results showed that *Fndc5* mutant mice had a much smaller ovarian size and lower ovarian mass index than WT mice (7.97 ± 1.65 μl vs 17.11 ± 2.62 μl, *P* < 0.05; 2.50 × 10^− 4^ ± 1.70 × 10^− 5^% vs 4.00 × 10^− 4^ ± 3.97 × 10^− 5^%, *P* < 0.05; respectively) (Fig. 4a-c). Apparent histological changes were also observed in the ovaries of *Fndc5* mutant mice (Fig. 4f). Mouse ovary serial sections stained with H&E were evaluated by counting the numbers of follicles in different stages, defined as primordial, primary, secondary, antral, or Graafian follicles. The ratios of secondary or antral follicles to primordial follicles and antral follicles to total follicles in *Fndc5* mutant mice were significantly lower than those in WT mice (26.8% vs 78.4%, *P* < 0.05; 7.2% vs 19.7%, respectively; P < 0.05) (Fig. 4d, e). These data indicate that irisin deletion exerted deleterious effects on ovary development, ovulation and luteinization.

#### Ovarian hormone level measurements

**Fig. 5:**
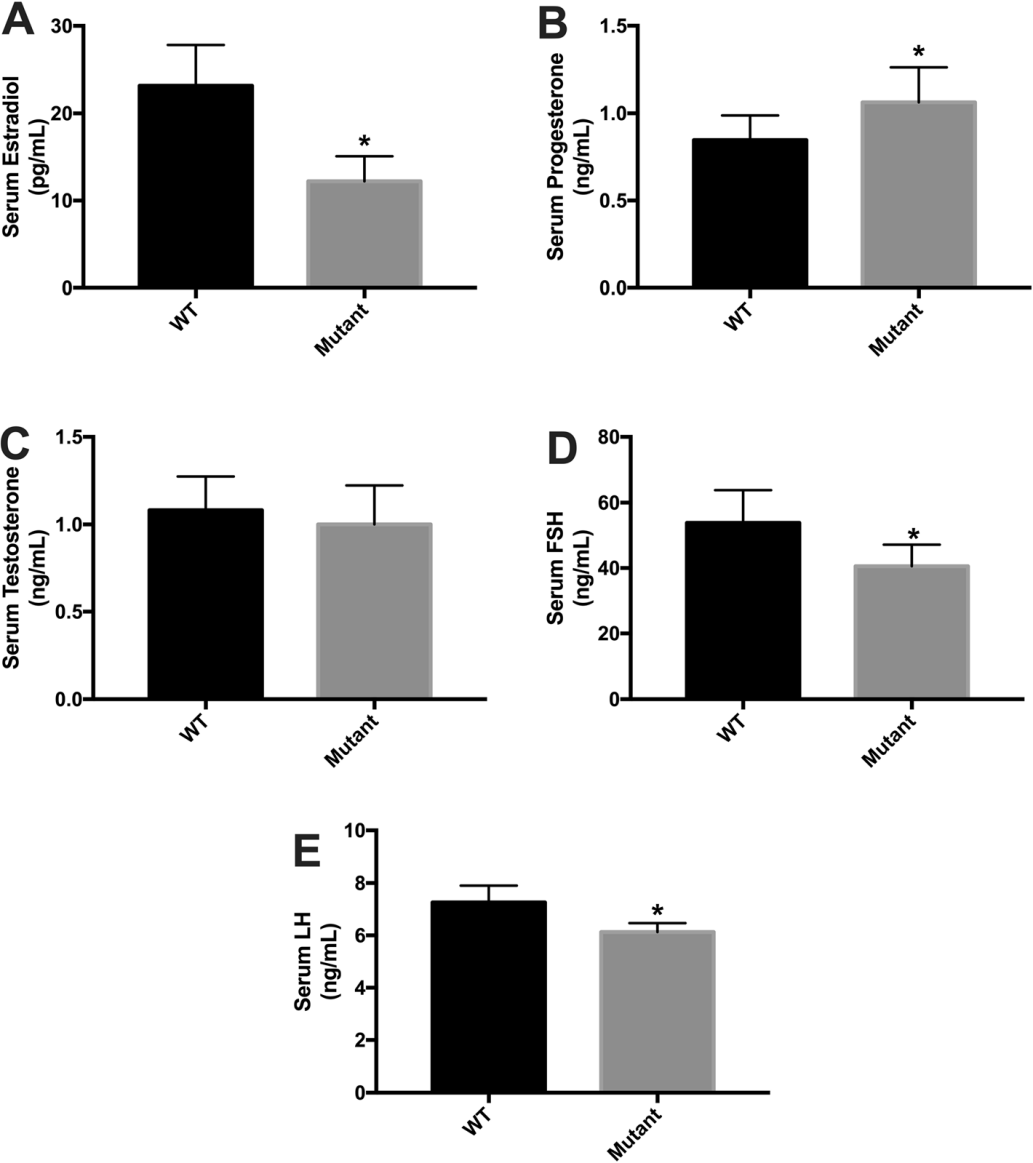
Hormone concentrations in *Fndc5* mutant mice (n = 10 per group). a: Estradiol (E_2_). b: Progesterone (P). c: Testosterone (T). d: Follicle-stimulating hormone (FSH). e: Luteinizing hormone (LH). *P < 0.05 compared to WT mice

**Fig. 6:**
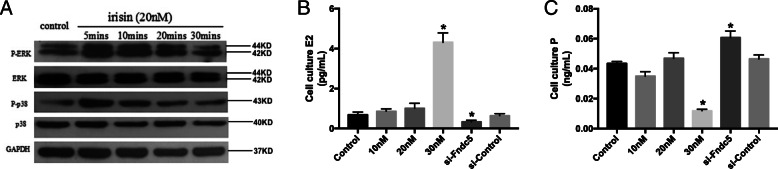
Effects of irisin on hormone production in KGN cells. Cells were incubated with increasing concentrations of irisin (10–30 nM) or si-Fndc5 (100 nM). a: Irisin stimulated the phosphorylation of the p38 and ERK proteins. b: The E_2_ concentration in the conditioned culture medium was measured by a specific radioimmunoassay. c: The progesterone (P) concentration was measured. Data are presented as the mean ± SD, (*n* = 3 per group); *P < 0.05 compared to the control group

The frequency, length and cytology of the estrous cycle reflect the hormonal milieu that maintains ovulatory function [17]. To evaluate ovarian function in irisin-deficient mice, the serum levels of E2, P and T were evaluated by ELISA. Compared with WT mice, the *Fndc5* mutant mice exhibited a significantly lower E2 concentration (12.20 ± 2.86 pg/ml VS 23.13 ± 4.86 pg/ml, *P* < 0.05) (Fig. 5a). However, irisin deletion increased the P level in *Fndc5* mutant mice compared to WT mice (1.06 ± 0.20 ng/ml vs 0.85 ± 0.14 ng/ml, *P* < 0.05, Fig. 5b). The testosterone level was comparable between *Fndc5* mutant and WT mice (Fig. 5c). In addition, FSH and LH levels were assessed. The data indicated that FSH and LH levels were decreased in *Fndc5* mutant mice compared to WT mice (106.84 ± 9.94 ng/ml vs 135.81 ± 22.83 ng/ml; 82.58 ± 15.35 ng/ml vs 106.76 ± 19.05 ng/ml, respectively; *P* < 0.05) (Fig. 5d, e). On the other hand, the effects of irisin were determined in vitro. We examined E2 and P concentrations in the culture medium of KGN cells. After KGN cells were treated with 10–30 nM irisin for 48 h, the level of E2 increased approximately 7-fold in the 30 nM group (4.32 ± 0.47 pg/ml vs 0.68 ± 0.15 pg/ml, *P* < 0.05) (Fig. 6b). However, the P concentration was decreased by 10 nM irisin stimulation when compared to control stimulation (0.012 ± 0.001 ng/ml vs 0.043 ± 0.002 ng/ml, P < 0.05) (Fig. 6c). In addition, KGN cells were incubated with siRNA (100 nM) for *Fndc5*, the E2 level decreased and P level increased compared to control group (0.34 ± 0.04 pg/ml vs 0.68 ± 0.15 pg/ml, P < 0.05; 0.06 ± 0.002 ng/ml vs 0.043 ± 0.002 ng/ml, P < 0.05; respectively).

#### Irisin regulated the expression of hormone-production-related proteins

**Fig. 7:**
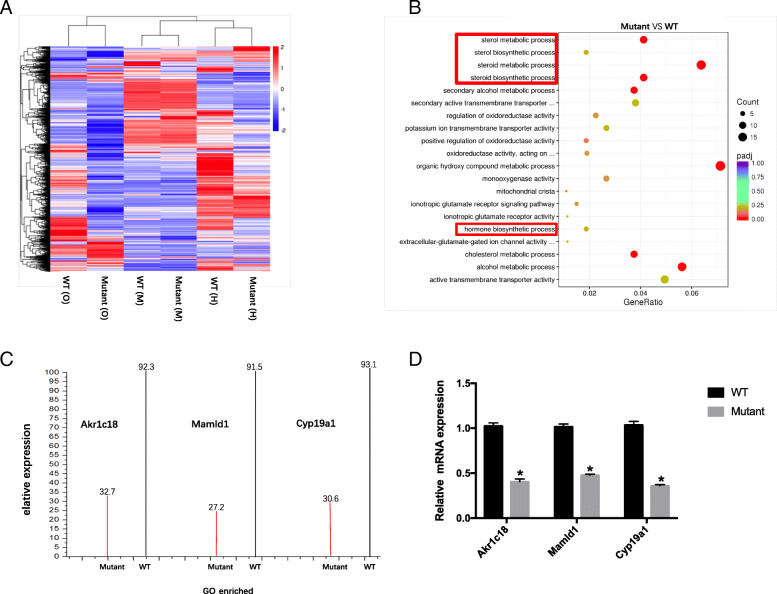
mRNA expression related to reproduction is downregulated in *Fndc5* mutant mice. a: The heat map for mRNA analyses of the ovaries, muscle and hypothalamus in WT and *Fndc5* mutant mice. b: The downregulated mRNAs in *Fndc5* mutant mice are associated with steroid hormone synthesis. c: The different MS/MS spectra of *Akr1c18*, *Mamld1* and *Cyp19a1* in *Fndc5* mutant and WT mice are shown. d: qRT-PCR results for the ovaries confirmed the decreased expression of *Akr1c18*, *Mamld1* and *Cyp19a1* in *Fndc5* mutant mice compared with WT mice. (n = 5 biologically independent *Fndc5* mutant or WT mice). Data are presented as the mean ± SD; *P < 0.05 compared to WT mice

To determine the molecular mechanism involving irisin in reproduction, we analyzed the transcriptional profiles of the ovaries in *Fndc5* mutant and WT mice through a transcriptome-sequencing assay. A total of 2217 genes were quantified, of which 1106 genes were differentially expressed (P < 0.05), including 911 upregulated and 195 downregulated genes (Fig. 7a).

Among the downregulated genes, 28 are involved in steroid hormone biosynthesis, which is crucial for reproduction (Fig. 7b). According to the literature, three of these genes, namely, Aldo-keto reductase family 1-member C1 (*Akr1c18*), mastermind-like domain containing 1 (*Mamld1*) and P450 aromatase (*Cyp19a1*), are directly related to the biosynthesis of sex hormones [18]. Gene Ontology (GO) enrichment results showed that *Akr1c18*, *Mamld1* and *Cyp19a1* expression decreased by 59.6, 64.3 and 62.5%, respectively (Fig. 7c). Furthermore, we confirmed that the mRNA levels of *Akr1c18*, *Mamld1* and *Cyp19a1* in *Fndc5* mutant mouse ovaries were approximately 57.6, 52 and 64% lower than those in WT mouse ovaries, respectively (Fig. 7d).

**Fig. 8:**
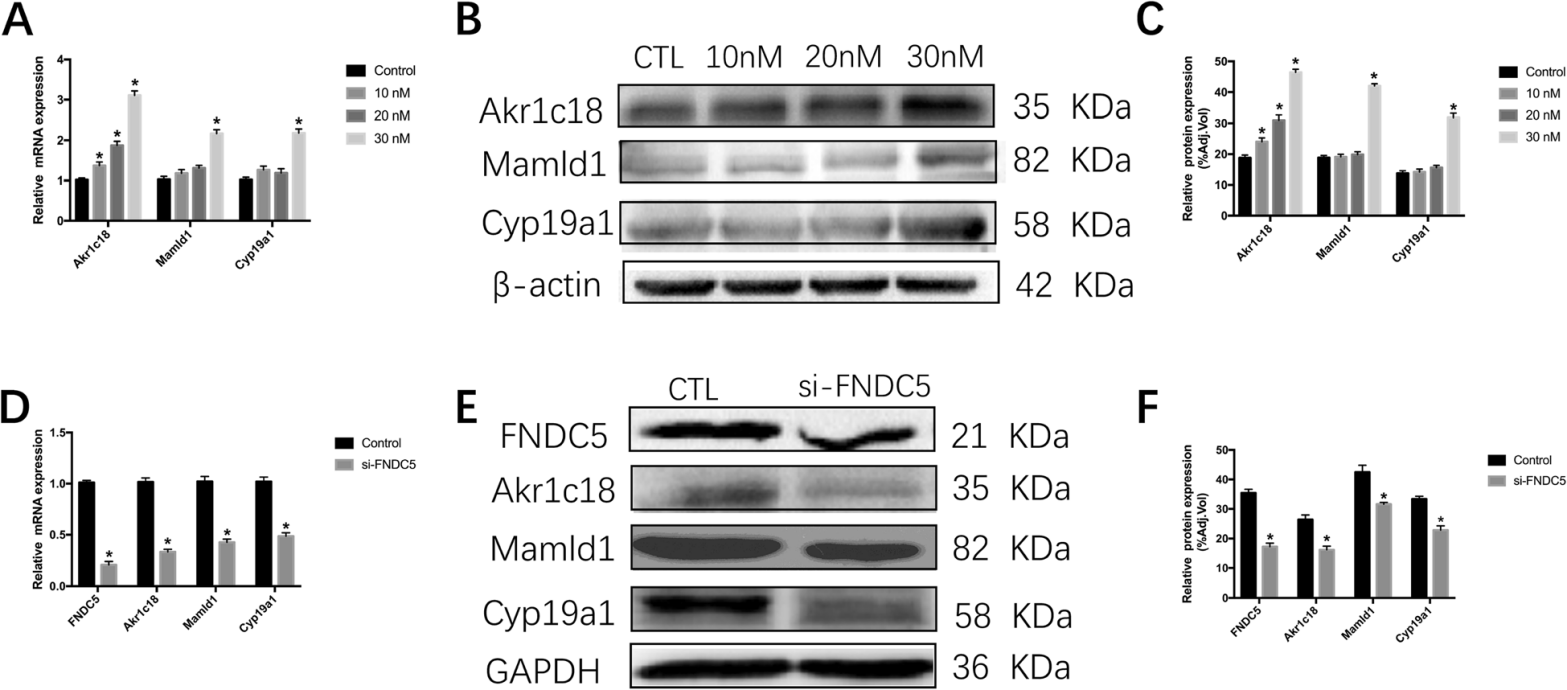
The effects of irisin on the expression of *Akr1c18*, *Mamld1* and *Cyp19a1* in vitro. KGN cells were cultured in the absence or presence of irisin (10–30 nM) for 48 h. a: Irisin increased the mRNA levels of *Akr1c18*, *Mamld1* and *Cyp19a1* (data from qRT-PCR). The mRNA levels were normalized to the *GAPDH* mRNA levels. Data are presented as the mean ± SD (n = 3). b, c: After exposure of KGN cells to the indicated concentrations of irisin for 72 h, the protein levels of *Akr1c18*, *Mamld1* and *Cyp19a1* were determined by Western blot analysis, and protein expression levels were quantified. d: mRNA was detected 48 h after siRNA transfection. e, f: *Fndc5*, *Akr1c18*, *Mamld1* and *Cyp19a1* expression at the protein level was detected 72 h after transfection (n = 3); *P < 0.05 compared to the control group

To confirm the effects of irisin on the expression of these three genes, KGN cells were exposed to serial concentrations (10–30 nM) of irisin for 48 h. The results suggested that the mRNA levels of *Akr1c18*, *Mamld1* and *Cyp19a1* were significantly higher in the 30 nM irisin-treated group than in the control group, as the 30 nM irisin-treated group exhibited 3.1-fold upregulation of *Akr1c18* gene expression, 2-fold upregulation of *Mamld1,* and 2.1-fold upregulation of *Cyp19a1* (Fig. 8a). Consistently, the effects of irisin were confirmed at the protein level (Fig. 8b, c). In addition, to test whether downregulation of irisin expression contributed to the expression of *Akr1c18*, *Mamld1* and *Cyp19a1*, siRNAs specific for *Fndc5* were designed and transfected into KGN cells. We tested a total of three siRNAs, and si-FNDC5#1 significantly knocked down the *Fndc5* mRNA level by ~ 80% (Fig. 8d). This siRNA also substantially reduced the expression of *Akr1c18* (~ 65%), *Mamld1* (~ 53%) and *Cyp19a1* (~ 49%) at the mRNA and protein levels (Fig. 8e, f).

## Discussion

Irisin regulates body energy expenditure by turning white adipose tissue into brown-like adipose tissue [3]. Binay et al. showed that elevated irisin levels were correlated with increased BMI, waist/hip ratio, fat percentage, and fat mass [19]. In this study, irisin deficiency were associated to increased mortality, and reduced body weight in female mice. Our results showed that the irisin deficient mice had higher GH levels but lower IGF-1 levels in serum. The biological actions of GH are pleiotropic and include growth promotion, energy mobilization, gonadal development, appetite, and social behavior [20]. IGF-1 is a growth-promoting peptide that shares significant structural homology with insulin. GH and IGF-1 are both anabolic hormones that take part in embryonic development and postnatal growth [21, 22]. GH induces the synthesis of IGF-1, and IGF-1 exerts feedback control on GH release [23]. Lian et al. showed that removal of irisin can increase the GH transcript level in pituitary cells [24]. Our results consistently showed that the expression of GH was increased in mRNA level in irisin deficient mice (Supplementary Fig. S1), which had an elevated GH level in serum. Previous studies have indicated that irisin is positively correlated with IGF-1 in humans and irisin dose dependently increases IGF-1 expression in primary human skeletal muscle cells [25, 26]. These results are consistent with our findings that the irisin deficient mice had lower IGF-1 concentration in serum. Renavillr et al. showed that the prolonged exposure to GH results in a reduced number of high-affinity growth hormone receptors (GHRs), resulting in GH resistance and IGF-1 reduction, showing anti-insulin effects [27, 28]. In addition, the increased mRNA level of IGF-binding proteins (IGFBPs), may be partly responsible for the decreased IGF-1 level in irisin deficient mice. IGFBPs in the plasma bind to IGF-1 to promote and/or inhibit its actions. IGFBP-2 and IGFBP-4 are known to inhibit IGF-1 activity in metabolism [29]. Our results showed that the *Fndc5* mutant mice had increased IGFBP-2, IGFBP-3, and IGFBP-4 levels at the transcript level (Supplementary Fig. S1). Additionally, our previous study showed that the IL-6 and TNF-α level were higher in mice lacking irisin [30]. Therefore, the derangement GH/IGF-1 axis activity, and the elevated proinflammatory cytokines were likely associated to the increased mortality, and poor growth and development in irisin deficient mice.

The effects of irisin on the reproductive system remain vague. In women, the E_2_ concentration is positively correlated with the irisin level [25]. However, in the case of poor estrogen production after ovariectomy (OVX), irisin levels are ~ 25% higher in OVX rats than in control rats [31]. In addition, irisin can stimulate LH production in pituitary mPitA12 cells and promote E_2_ production in ovary granulosa cells [10]. Suat et al. demonstrated that irisin played a role in reducing the testosterone level by suppressing LH and FSH secretion in male rats [11]. These discrepancies may be related to species, sex, irisin concentration and so on.

To our knowledge, our study represents the first report to demonstrate the association between irisin and reproduction in female mice. In our study, the mice lacking irisin showed disordered steroid hormone levels, reduced ratio of antral follicles and irregular estrous cycle, which related to a decrease fertility in female mice. Our results indicated that irisin had positively effects on expression of *Akr1c18*, *Mamld1* and *Cyp19a1* in ovaries. *Akr1c18* is the gene encoding 20α-hydroxysteroid dehydrogenase (20α-HSD), which converts progesterone into the inactive metabolite 20α-hydroxyprogesterone (20α-OHP) [32]. Piekorz et al. showed that *Akr1c18* deletion in mice leads to persistent progesterone production and subsequent parturition failure [32, 33]. Our results showed a decreased expression of *Akr1c18* in irisin deficient mice, which had hyperlutemia with reduced clearance of progesterone. Miyado et al. found that *Akr1c18* expression was regulated by *Mamld1* in murine Leydig tumor cells (MLTC1) [18]. Consistently, our results showed *Akr1c18* expression was positively associated with *Mamld1* in KGN cells. *Mamld1* has been demonstrated to be related to testosterone production in MLTC1 cells, and has been found to be a causative gene in inordinate of sex development (DSDs) with hypospadias as a salient clinical phenotype [34, 35]. P450 aromatase (*Cyp19a1*) is known to be a rate-limiting enzyme in estrogen biosynthesis [36]. The decrease expression of *Cyp19a1* in irisin deficient mice was associated with lower E_2_ production in vivo. The effects of irisin on *Akr1c18*, *Mamld1* and *Cyp19a1* were also confirmed in vitro. Irisin could promote the expression of *Akr1c18*, *Mamld1* and *Cyp19a1* in both mRNA and protein levels, with the increased E_2_ level and decreased P level in KGN culture medium. However, the stimulating effects of irisin could inhibit by siRNA-*Fndc5* treatment.

In mammals, reproduction is regulated by the hypothalamus-pituitary-gonadal (HPG) axis. The hypothalamus produces GnRH, which regulates the synthesis of LH and FSH, and these two hormones are secreted from the anterior pituitary. Gonadotropins, in turn, act on the gonads (the ovaries or testes) to stimulate gonadal development [2]. E_2_ and P are the co-mediator of the negative feedback mechanism [37, 38]. In the present study, the mice lacking irisin showed lower FSH and LH levels, which were likely due to two reasons. First, Quan et al.^38^ reported that irisin was effective in stimulating both FSHβ and LHβ mRNA expression in pituitary cells. Thus, irisin deficient mice likely have direct impact on FSH and LH synthesis. Second, progesterone is the primary modulator of GnRH pulse frequency slowing in women. For example, the LH (and by inference GnRH) pulse frequency slows as the progesterone level increases in the luteal phase [39, 40]. Therefore, the hyperlutemia in irisin deficient mice indirectly inhibited FSH and LH production through negative feedback. Then, the lower FSH and LH levels have several adverse effects on reproduction. For example, the decreased FSH level influenced the development of antral follicles, aggravating the decrease in E_2_ production. The deficiency of LH surge, resulted in oocyte maturation and ovulation disorder. In addition, the decreased E2 level ad increased P level were associated to the irregular estrous cycle in mice lacking irisin. The prolonged proestrus time and shortened estrus time in *Fndc5* mutant mice were related to the poor fertility.

Polycystic ovary syndrome (PCOS) is considered one of the most frequent endocrinopathies affecting women of child-bearing age. It is associated not only with reproductive problems characterized by hyperandrogenemia and chronic oligo- or anovulation but also metabolic alterations leading to obesity and obesity-related disorders diseases [41, 42]. Insulin resistance (IR) is a factor linking PCOS and metabolic syndrome [43]. Several studies have examined the association between irisin and blood glucose levels and IR in subjects. To date, the results about irisin and PCOS are controversial. Most studies have reported higher irisin levels in polycystic ovary syndrome (PCOS) women that controls [44–46], whereas some other studies reported similar [47, 48] or lower [49] circulating irisin levels in PCOS women than controls. Li et al. [44] showed metformin treatment could decrease circulating irisin in PCOS patients together with IR. Further studies in cautiously selected populations and matched controls are needed.

In this study, the *Fndc5* mutant mice were generally normal in appearance, but there were some problems with internal functions, such as poor development and fertility. We also assessed glucose metabolism and bone metabolism, which will be described in other articles. In addition, we edited the *Fndc5* gene to generate an irisin-deficient model. According to our method, although there was certainly no irisin in the animal models, the functions of other parts of *Fndc5* were also affected. Therefore, this animal model is not an ideal model. Due to the limitation of technology, we could not obtain the ideal model that had irisin deleted without affecting other functions of *Fndc5*. Thus, hopefully, the relevant research group can provide us with this ideal model. Additionally, we need to conduct further research in reproduction to explore the relationship between irisin and disease.

## Conclusions

In summary, we have demonstrated for the first time that irisin deficiency was related to disordered endocrinology metabolism in mice. The irisin deficient mice showed increased mortality, poor growth and development, and decreased fertility. Irisin likely have effects on the expressions of *Akr1c18*, *Mamld1* and *Cyp19a1* in ovary, regulating the steroid hormone production. This study provides novel insights into the potential role of irisin in mammalian growth and reproduction.

## Supplementary Information

**Additional file 1: Figure S1.** The different MS/MS spectra of *Akr1c18*, *Mamld1* and *Cyp19a1* in *Fndc5* mutant and WT mice are shown GH, IGF-1, IGFBP-2, IGFBP-3 and IGFBP-4 in *Fndc5* mutant and WT mice are shown.

## Data Availability

All the supporting data are availability.

## Abbreviations

Fndc5: fibronectin type III domain-containing protein 5
FSH: follicle-stimulating hormone
LH: luteinizing hormone
HPG: hypothalamus-pituitary-gonadal
IGF-1: insulin-like growth factor I
ERK: extracellular regulated protein kinases
KGN: ovarian granulosa
IGFBPs: IGF-binding proteins
GnRH: gonadotropin-releasing hormone
