# Supplementary material for: Irisin deletion induces a decrease in growth and fertility in mice
Source: Reprod Biol Endocrinol. 2021 Feb 13;19:22. doi: 10.1186/s12958-021-00702-7 (PMC7881587; doi:10.1186/s12958-021-00702-7)

**Supplementary material**

1. The full western blots of Figure 8.

(1)The expression of proteins in KGN cell by irisin treatment.

**
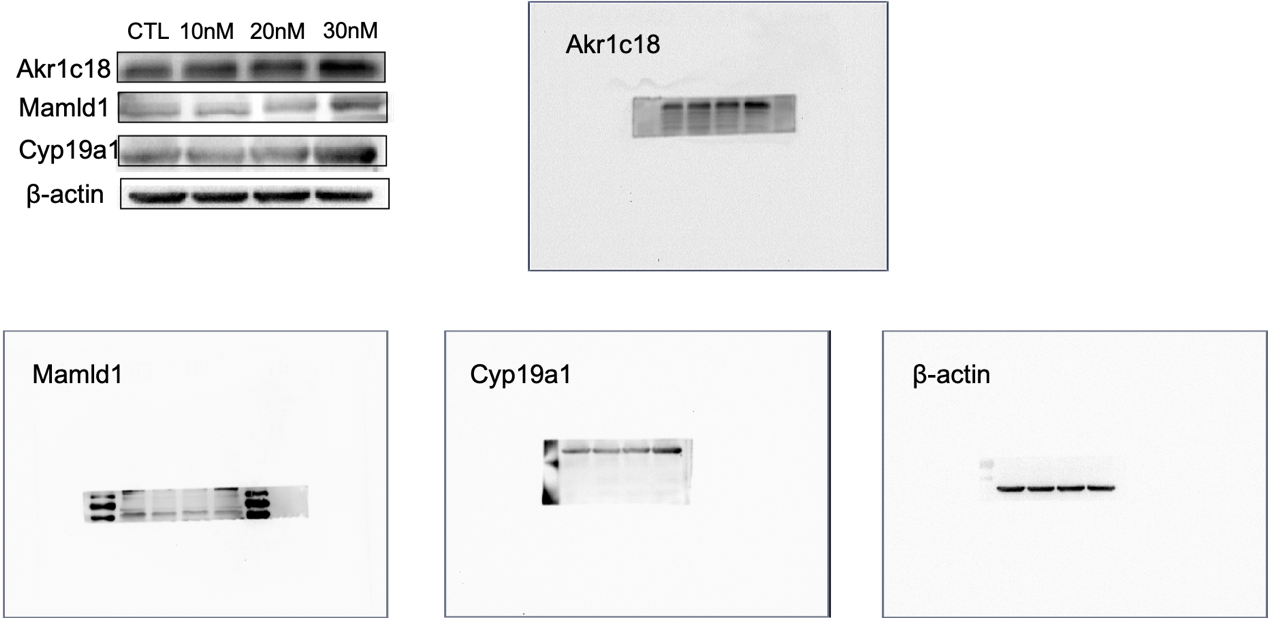
**

(2) The expression of proteins in KGN cell by Fndc5-siRNA treatment.

**
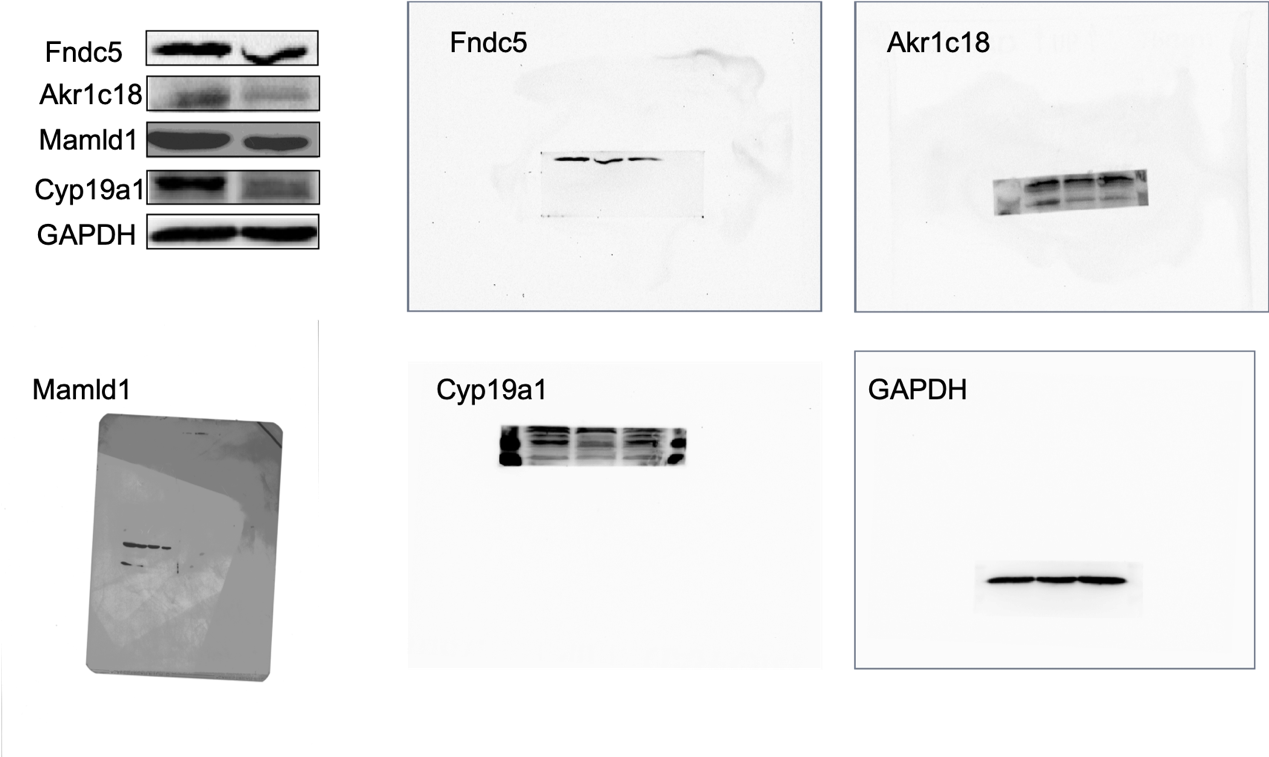
**

2. Supplementary Figure 1.


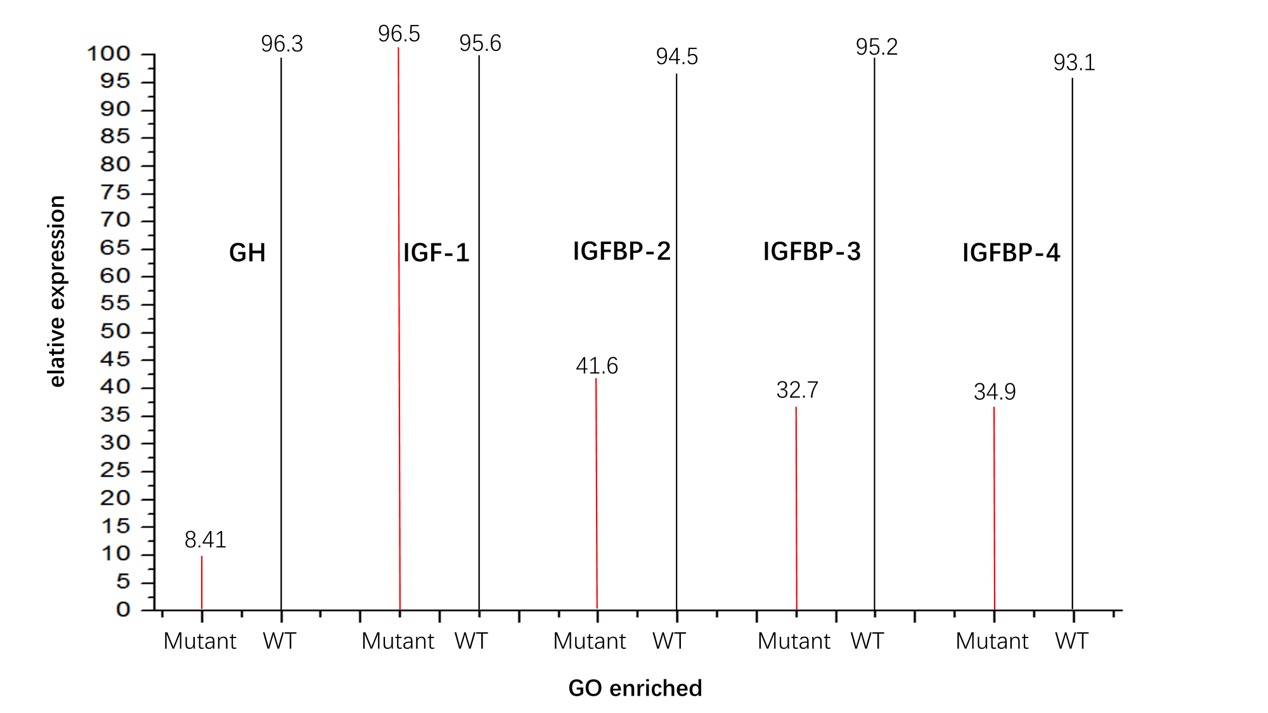

Supplement: Supplementary file 1 — Additional file 1: Figure S1. The different MS/MS spectra of Akr1c18, Mamld1 and Cyp19a1 in Fndc5 mutant and WT mice are shown GH, IGF-1, IGFBP-2, IGFBP-3 and IGFBP-4 in Fndc5 mutant and WT mice are shown. [file 12958_2021_702_MOESM1_ESM.docx]
